# Implementation of an FMEA-guided quality-improvement workflow is associated with improved setup accuracy in lung stereotactic body radiotherapy

**DOI:** 10.3389/fonc.2026.1860664

**Published:** 2026-07-22

**Authors:** Zunhao Wen, Zhiwei Liu, Weizhi Li, Weiwei Wu, Hui Yin

**Affiliations:** 1 Department of Radiation Oncology, Ganzhou Cancer Hospital, Ganzhou, China; 2 Department of Medical Imaging, Ganzhou Cancer Hospital, Ganzhou, China

**Keywords:** failure mode and effects analysis (FMEA), lung cancer, risk management, setup errors, stereotactic body radiotherapy (SBRT)

## Abstract

**Background:**

Lung stereotactic body radiotherapy (SBRT) requires highly reproducible workflow execution because of steep dose gradients, respiratory motion, and narrow treatment margins. This study evaluated the workflow effectiveness and process-safety impact of an FMEA-guided quality-improvement workflow for lung SBRT.

**Methods:**

This single-center historical-control study included 128 patients treated with lung SBRT. Sixty-three patients treated under conventional quality-control procedures served as the control group, and 65 patients managed after implementation of an FMEA-guided quality-improvement workflow served as the FMEA-guided group. The workflow included risk identification, targeted corrective measures, respiratory coaching, dual verification, immobilization optimization, and enhanced equipment quality control. RPN changes were analyzed using the Wilcoxon signed-rank test. CBCT-derived setup deviations were assessed using patient-level median absolute couch correction values and fraction-level linear mixed-effects models. Workflow-related adverse events were compared using Fisher’s exact test.

**Results:**

Eleven high-risk failure modes were identified. The overall median residual RPN was lower than the initial RPN [39.0 (IQR, 26.0–46.5) vs 177.5 (IQR, 165.0–209.0); *p* = 0.003]. The setup-error analysis included 374 CBCT registrations from 63 control patients and 388 registrations from 65 patients in the FMEA-guided group. Patient-level median absolute couch correction values were smaller in the FMEA-guided group, and fraction-level mixed-effects models confirmed significantly smaller deviations across all six translational and rotational dimensions. Workflow-related adverse events decreased from 11.11% to 3.08%, but the difference was not statistically significant (RR = 0.277, 95% CI, 0.060–1.282; *p* = 0.093).

**Conclusion:**

Implementation of an FMEA-guided quality-improvement workflow was associated with reduced residual RPN scores and smaller CBCT-derived setup deviations in lung SBRT. Workflow-related adverse events decreased numerically but did not reach statistical significance. These findings support the value of proactive, closed-loop workflow risk management, while further prospective multicenter validation is warranted.

## 1 Introduction

Stereotactic body radiotherapy (SBRT) has become a cornerstone treatment for early-stage non-small cell lung cancer (NSCLC) and pulmonary metastases. By delivering ablative radiation doses in a limited number of fractions, SBRT achieves excellent local control and survival outcomes. However, this high-dose, hypofractionated approach also imposes stringent requirements on treatment precision and procedural safety (1–3).

The SBRT workflow for lung cancer is inherently complex, involving multiple interdependent steps, including patient immobilization, computed tomography (CT) simulation, target delineation, treatment planning, and treatment delivery. Errors or deviations at any stage may propagate through the workflow, leading to clinically significant consequences such as abnormal dose distribution, geographic misses, or severe toxicity to normal tissue (4). Notably, previous studies have reported that up to 76% of radiotherapy-related incidents occur prior to treatment delivery, with the majority attributable to human error and process deficiencies (5).

Despite ongoing advancements in radiotherapy technology, conventional quality control (QC) strategies remain predominantly focused on equipment performance and dosimetric verification. While these measures are essential, they may not fully address process-related vulnerabilities arising from complex multidisciplinary workflows. In fact, more than 70% of medical errors in clinical practice have been linked to failures in workflow transitions and human factors (6). In the context of lung SBRT, where precision margins are minimal, even minor procedural deviations, such as suboptimal respiratory training or ambiguous fiducial marker identification, can lead to drastic fluctuations in dose distribution.

Failure Mode and Effects Analysis (FMEA) is a prospective risk assessment tool designed to systematically identify and prioritize potential failure modes within complex processes. By quantifying the frequency of occurrence, severity, and detectability, FMEA generates a Risk Priority Number (RPN) to guide targeted risk mitigation. Initially developed in high-reliability industries such as aviation and automotive engineering, FMEA has been increasingly introduced into healthcare as an effective framework for enhancing patient safety and process quality (7). Unlike previous studies that mainly reported theoretical RPN scoring or general radiotherapy risk assessment, the present study attempted to close the FMEA loop by linking lung SBRT-specific high-risk failure modes to targeted corrective measures and measurable process endpoints, including CBCT-derived six-dimensional setup deviations and recorded workflow-related events. Given the historical-control design, the study was intended to evaluate the implementation effect of an FMEA-guided quality-improvement workflow rather than to establish definitive causal clinical efficacy.

## 2 Materials and methods

### 2.1 Patient selection and characteristics

This was a single-center, historically controlled quality-improvement study comprising a retrospectively identified control cohort and prospective implementation of an updated workflow in the intervention cohort. A total of 128 patients with lung cancer who underwent SBRT at the Department of Radiation Oncology, Ganzhou Cancer Hospital, between July 2023 and February 2026 were enrolled. The inclusion criteria were as follows: (1) pathologically or radiologically confirmed NSCLC or lung metastasis; (2) Karnofsky Performance Status (KPS) score≥70; (3) adequate pulmonary function to cooperate with 4DCT scanning and respiratory coaching; and (4) complete clinical and follow-up records. Exclusion criteria included: (1) severe systemic diseases (e.g., severe cardiac, hepatic, or renal dysfunction); (2) contraindications to radiotherapy or active severe infections; and (3) inability to comply with the SBRT workflow. The study protocol was reviewed and approved by the Ethics Committee of Ganzhou Cancer Hospital [(2026) Kelunshen No.132]. The control cohort was retrospectively identified from routinely collected clinical records, whereas the intervention cohort was managed under an updated institutional quality-improvement workflow. Because the intervention represented refinements to routine lung SBRT quality-control procedures and did not involve additional experimental treatment, the requirement for additional study-specific written informed consent was waived by the Ethics Committee. All patient data were anonymized before analysis.

Patients were categorized into two groups according to the risk management strategy. The control group comprised 63 patients treated under conventional quality control (QC) protocols between July 2023 and December 2024, including 45 males (71.43%) and 18 females (28.57%), with a mean age of 63.2 ± 10.1 years. The FMEA-guided group included 65 patients prospectively managed using the Failure Mode and Effects Analysis (FMEA) framework between January 2025 and February 2026, including 49 men (75.38%) and 16 women (24.62%), with a mean age of 64.4 ± 11.0 years.

Baseline and treatment-related variables included sex, age, body mass index, tumor laterality, tumor origin, central versus peripheral lesion location, gross tumor volume, prescription dose, dose per fraction, number of treatment fractions, respiratory-motion management, and the number of CBCT-guided treatment fractions (**Table 1**). The setup-error analysis included 374 fraction-level CBCT registrations from 63 patients in the control group and 388 registrations from 65 patients in the FMEA-guided group. All patients underwent free-breathing 4DCT simulation for respiratory-motion assessment and internal target volume determination. Treatment was delivered under free breathing for most patients, whereas respiratory-gated delivery was selectively used in patients for whom the treating team considered respiratory-motion control clinically necessary. Respiratory-gated treatment was used in 11 of 63 patients in the control group and 10 of 65 patients in the FMEA-guided group. The distribution of respiratory-motion management strategies did not differ significantly between the two cohorts (*p* = 0.814). The immobilization approach and online image-guidance criteria were consistent during the control and FMEA-guided study periods.

**Table 1:** Baseline characteristics of 128 patients with lung cancer.

Characteristic	Control group (n=63)	FMEA-guided group (n=65)	Test	*P* value
Gender, n (%)				
Male	45(71.43)	49(75.38)	Fisher’s exact test	0.691
Female	18(28.57)	16(24.62)		
Age, years	63.2 ± 10.1	64.4 ± 11.0	*t* test	0.522
BMI, kg/m^2^	22.35 ± 3.20	22.81 ± 3.25	*t* test	0.417
Tumor Laterality, n (%)				
Left lung	32(50.79)	34(52.31)	Fisher’s exact test	1.000
Right lung	31(49.21)	31(47.69)		
Tumor Origin, n (%)				
Primary	17(26.98)	18(27.69)	Fisher’s exact test	1.000
Metastatic	46(73.02)	47(72.31)		
Tumor Location, n (%)				
Peripheral	42 (66.7%)	40 (61.5%)	Fisher’s exact test	0.584
Central	21 (33.3%)	25 (38.5%)		
GTV volume, cm³	17.5 (11.9–27.7)	15.6 (6.5–24.4)	Mann–Whitney U test	0.129
Prescription dose, Gy	50.0 (40.0–55.0)	50.0 (42.0–56.0)	Mann–Whitney U test	0.219
Dose per fraction, Gy	6.0 (5.0–8.0)	6.0 (5.0–8.0)	Mann–Whitney U test	0.179
Number of fractions	8.0 (5.0–10.0)	8.0 (5.0–10.0)	Mann–Whitney U test	0.994
CBCT registrations per patient	6(4-7)	6(4-7)	Mann–Whitney U test	0.971
Total CBCT registrations, n	374	388	Descriptive	–
Respiratory-motion management, n (%)				
Free breathing	52 (82.5%)	55 (84.6%)	Fisher’s exact test	0.814
Respiratory-gated treatment	11 (17.5%)	10 (15.4%)		

Data are presented as mean ± SD, median (IQR), or n (%), as appropriate. Age and BMI were compared using the independent-samples t test. GTV volume, prescription dose, dose per fraction, number of fractions, and CBCT registrations per patient were compared using the Mann–Whitney U test. Categorical variables were compared using Fisher’s exact test. Total CBCT registrations represent the number of fraction-level image-guidance records included in the analysis and were summarized descriptively without hypothesis testing. BMI, body mass index; CBCT, cone-beam computed tomography; FMEA, failure mode and effects analysis; GTV, gross tumor volume; IQR, interquartile range; SBRT, stereotactic body radiotherapy.

During the study period, the hardware of the VitalBeam linear accelerator and Eclipse 15.6 treatment planning system remained consistent without major upgrades. In addition, the multidisciplinary team—including radiation oncologists, medical physicists, and radiation therapists—remained stable, with an average clinical experience of more than 5 years, thereby limiting variability attributable to major equipment upgrades or personnel turnover, although time-dependent improvement in staff proficiency could not be excluded.

### 2.2 Equipment and technical parameters

Treatment was delivered using a Varian VitalBeam linear accelerator equipped with an On-Board Imager (OBI) system, Respiratory Gating for Scanners (RGSC), and Cone-Beam CT (CBCT) modules. The patients were positioned in the supine position and immobilized using a dedicated stereotactic body frame combined with a vacuum cushion and thermoplastic mask. A 4DCT scan (slice thickness: 2.5 mm) was performed. Target volumes, including Gross Tumor Volume (GTV), Internal Target Volume (ITV), and Planning Target Volume (PTV), were sequentially delineated using the Eclipse treatment planning system. SBRT plans were optimized with 6 MV flattening filter-free (FFF) X-rays, typically delivered via 2–4 arcs at a dose rate of 1400 cGy/min. Prescription doses ranged from 5 to 12 Gy per fraction over 3 to 10 fractions, ensuring that 95% of the PTV received the prescribed dose. Before each treatment fraction, CBCT was acquired and registered to the planning reference images for setup verification and online couch correction. Under the institutional online correction protocol, patient repositioning and repeat CBCT verification were required when the translational deviation exceeded 3 mm in any direction or the rotational deviation exceeded 2°.

### 2.3 Risk management methods

#### 2.3.1 Control group

The control group followed conventional radiotherapy QC procedures. These included routine equipment maintenance, weekly accelerator output dose calibration, and verification of CBCT isocenter and image quality. Clinical protocols involved adherence to the “three checks and seven verifications” system, verification of patient information and treatment plan parameters, and verbal instructions for radiotherapy precautions and respiratory cooperation. Internal staff training was conducted through experience-sharing sessions.

#### 2.3.2 FMEA-guided group

The intervention comprised an FMEA-guided, multicomponent quality-improvement workflow. FMEA was used to identify and prioritize high-risk workflow nodes, whereas the implemented corrective measures included process standardization, staff training, respiratory coaching, dual verification, immobilization improvement, gating-threshold optimization, and enhanced equipment quality control.

**Figure 1:**
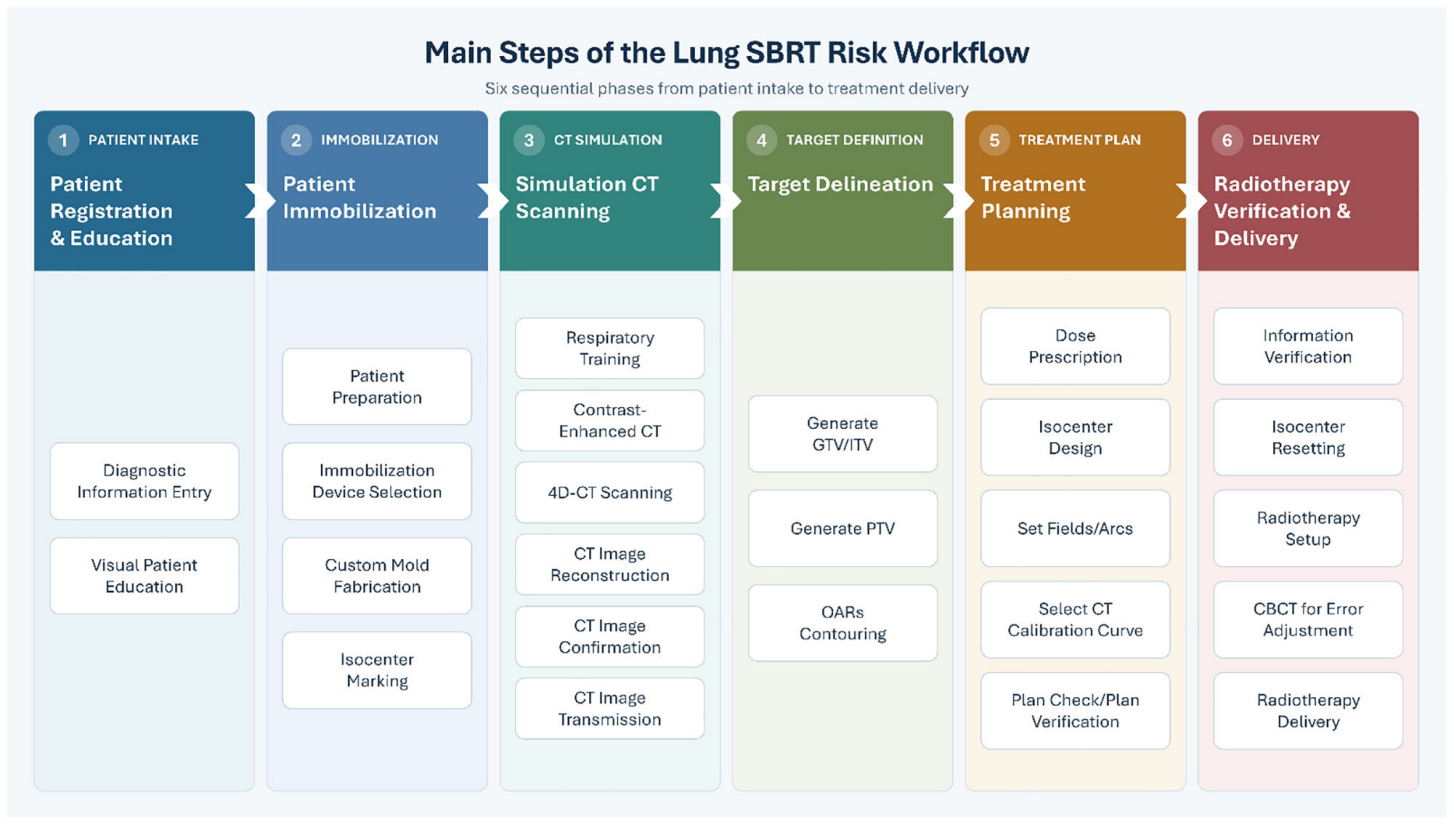
Main Steps of the Lung SBRT Risk Workflow Main steps of the lung SBRT risk workflow, comprising six sequential modules from patient registration to treatment verification and delivery. CBCT, cone-beam computed tomography; CT, computed tomography; GTV, gross tumor volume; ITV, internal target volume; OARs, organs at risk; PTV, planning target volume; SBRT, stereotactic body radiotherapy.

1. FMEA Team Formation: A multidisciplinary team was established, comprising two senior radiation oncologists, one medical physicist, four senior radiation therapists, and one oncology nurse specialist. All eight members completed theoretical training and practical exercises based on the AAPM TG-100 framework before participating in workflow mapping and risk scoring (8).

2. Process Mapping: Guided by current lung SBRT operational protocols and the TG-100 framework, the team deconstructed the workflow into six core modules: patient registration and education, immobilization, CT simulation, target delineation, treatment planning, and treatment delivery/verification. These modules were further subdivided into six main steps and 25 sub-steps, forming a structured visual framework to identify operational nodes and potential risk interfaces (**Figure 1**).

3. Prospective Identification of Failure Modes: Potential failure modes within the lung SBRT workflow were identified through structured brainstorming sessions and retrospective analysis of deviations observed in the control group. Each member independently scored the occurrence (O), severity (S), and detectability (D) of each failure mode on a 1–10 scale, following the TG-100 guidelines (**Table 2**) (8). All initial ratings were completed independently before group discussion. When the difference between individual ratings for a given O, S, or D component exceeded three points, the disagreement was reviewed during a multidisciplinary consensus meeting moderated by the senior radiation oncologists and medical physicist. The discussion was based on the TG-100 definitions and available institutional workflow records. After discussion, raters were allowed to revise their individual scores, and the retained ratings were used for subsequent summary and reliability analyses. For each rater and failure mode, the risk priority number was calculated as: RPN = O × S × D. Inter-rater reliability was assessed separately for O, S, D, and RPN using a two-way random-effects, absolute-agreement, average-measures intraclass correlation coefficient [ICC(2,k)] (9). For the initial assessment, the ICCs for O, S, D, and RPN were 0.952, 0.920, 0.952, and 0.758, respectively. An initial RPN >125 was used as a prespecified pragmatic criterion for prioritizing failure modes. Because the multiplicative RPN model may assign similar products to clinically different combinations of occurrence, severity, and detectability, RPN was not used as the sole prioritization criterion. Failure modes considered to have potentially serious consequences were additionally reviewed during multidisciplinary consensus meetings and were not excluded solely because their total RPN was below the threshold. Eleven high-risk failure modes were ultimately selected for targeted intervention.

**Table 2:** TG-100 report: risk level scoring criteria for failure modes.

Rank	Occurrence (O)		Severity (S)		Detectability (D)
	Qualitative	Frequency	Qualitative	Categorization	Estimated Probability of failure going undetected
1	Failure unlikely	0.01	No effect	–	0.01
2	Failure unlikely	0.02	Inconvenience	Inconvenience	0.2
3	Relatively few failures	0.05	Inconvenience	Inconvenience	0.5
4	Relatively few failures	0.1	Minor dosimetric error	Suboptimal plan or treatment	1
5	Relatively few failures	<0.2	Limited toxicity or tumor underdose	Wrong dose, dose distribution, location, or volume	2
6	Occasional failures	<0.5	Limited toxicity or tumor underdose	Wrong dose, dose distribution, location, or volume	5
7	Occasional failures	<1	Potentially serious toxicity or tumor underdose	Wrong dose, dose distribution, location, or volume	10
8	Repeated failures	<2	Potentially serious toxicity or tumor underdose	Wrong dose, dose distribution, location, or volume	15
9	Repeated failures	≤5	Possible very serious toxicity or tumor underdose	Very wrong dose, dose distribution, location, or volume	20
10	Failures inevitable	>5	Catastrophic	Very wrong dose, dose distribution, location, or volume	>20.0

4. Formulation and Implementation of Corrective Measures:

Targeted corrective measures were developed through multidisciplinary consensus according to the identified causes and risk characteristics of each high-risk failure mode. The interventions included standardized immobilization procedures, post-fabrication mold testing, standardized respiratory coaching and documentation, dual-person verification, standardized reference-image selection, mandatory manual review after automatic CBCT registration, gating-threshold verification, and enhanced equipment quality control (**Table 3**). Routine equipment maintenance, output calibration, patient-information verification, and general respiratory instructions were already used during the control period. The newly implemented measures primarily consisted of standardized respiratory-coaching records, post-fabrication immobilization checks, predefined dual-verification steps, standardized CBCT reference-image selection, and explicit re-positioning and re-verification criteria.

**Table 3:** Potential failure modes and interventions in the lung SBRT workflow.

Workflow phase	Main step	Failure mode	Cause of failure	Intervention measure
Immobilization	Mold fabrication	Inconsistent arm elevation; poor shoulder fit; lack of standard posture	Lack of operational standardization; insufficient therapist training.	Refine standardized SBRT immobilization protocols; conduct specialized therapist training.
	Mold fabrication	Thermoplastic mask shrinkage/deformation; poor vacuum cushion sealing.	Poor material resilience; lack of mold testing workflow.	Replace with high-resilience thermoplastic materials; establish post-fabrication sealing test; discard defective molds immediately.
	Isocenter marking	Inconsistent marking phase; uneven marking lines.	Lack of phase standardization; incomplete verification.	Standardize marking at the end-expiratory phase using 4DCT; use standardized markers; implement dual-person verification.
CT Simulation	Contrast-enhanced CT	Contrast injection induces patient anxiety, causing patient movement.	Inadequate patient understanding and pre-education.	Pre-treatment education on contrast injection by nurses; psychological counseling for anxious patients.
	CT scanning	Irregular breathing; inadequate respiratory training.	Absence of structured training process; lack of dedicated coaching records.	Produce respiratory training videos; dedicated nurse provides pre-treatment coaching and logs compliance.
	CT reconstruction	Failure to generate correct 4D reconstruction images.	Unfamiliar with equipment operation; lack of immediate image verification mechanism.	Collaborate with vendors for reconstruction training; establish immediate image verification workflow.
Verification & Delivery	Information verification	Discrepancy between treatment plan and physician’s prescription.	Over-reliance on automatic registration; non-standardized registration methods.	Adhere to CBCT registration guidelines; mandate manual review post-auto-registration; re-register if out of tolerance.
	Information verification	Inconsistent reference images.	Lack of criteria for reference image selection; high operational arbitrariness.	Establish lung SBRT reference image selection criteria; integrate into operational checklist.
	CBCT	Registration misalignment.	Setup errors uncorrected; respiratory motion compromises precision.	Utilize respiratory gating to minimize motion artifacts; reposition and re-verify if registration deviation > 3mm.
	Treatment delivery	Beam output dose deviation.	Irregular equipment QC; delayed maintenance.	Weekly inspections by engineering department; daily beam output checks by physicists; immediate halt for maintenance upon failure.
	Treatment delivery	Discrepancy between breathing patterns during treatment and simulation.	Gating thresholds uncalibrated; abnormal breathing unmanaged.	Recalibrate gating thresholds prior to delivery; pause treatment and retrain patients exhibiting abnormal breathing.

5 Residual-Risk Reassessment: Between January 2025 and February 2026, the optimized workflow was applied to the intervention cohort. After implementation, the same eight FMEA team members independently reassessed O, S, and D for the same 11 failure modes using the same TG-100 criteria and disagreement-resolution procedure. For each rater and failure mode, residual RPN was recalculated as O × S × D.

Residual-risk reassessment was not blinded because the raters were aware of the corrective measures that had been implemented. For the residual assessment, the ICCs for O, S, D, and RPN were0.770, 0.963, 0.772, and 0.513, respectively. Initial and residual O, S, D, and RPN values were summarized as median and interquartile range across the eight raters. Detailed rater-level values and component-level summaries are provided in **Supplementary Table 1**, including separate Initial_ratings and Residual_ratings worksheets. Because residual scoring was performed by the same unblinded team, changes in RPN were interpreted as changes in a semi-quantitative process-risk indicator rather than as an independent clinical endpoint.

### 2.4 Outcome measures

The efficacy was evaluated across three dimensions. First, the difference between the initial and residual RPN was calculated to quantify the theoretical reduction in risk. Second, for setup error analysis, CBCT setup data were retrospectively extracted for the control group and prospectively collected for the FMEA-guided group. Deviations across three translational axes (vertical [Vrt], longitudinal [Lng], lateral [Lat]) and three rotational axes (rotation [Rtn], Pitch, Roll) were compared. The reported six-dimensional setup deviations represented absolute couch correction values derived from online CBCT image registration before treatment delivery, rather than residual post-correction errors. For each treatment fraction, automatic registration was followed by manual review according to institutional lung SBRT registration criteria. Translational and rotational correction values were extracted as fraction-level setup-deviation records. Third, Workflow-related adverse events were defined as process deviations occurring during simulation, planning, image verification, or treatment delivery that required corrective action, additional verification, repeat imaging or simulation, replanning, treatment interruption or delay, or formal incident documentation. Events were identified from treatment records, quality-control documentation, and institutional workflow-event records using the same predefined event categories for both cohorts. The primary endpoint was the occurrence of at least one workflow-related adverse event during a patient’s treatment course. Each patient was counted once in the primary analysis, whereas individual event categories were summarized descriptively.

### 2.5 Statistical analysis

Statistical analyses were performed using SPSS version 26.0 (IBM Corp., Armonk, NY, USA) and R version 4.3.2 (R Foundation for Statistical Computing, Vienna, Austria). Continuous variables were tested for normality using the Shapiro–Wilk test and are presented as mean ± standard deviation or median (interquartile range), as appropriate. Between-group comparisons were performed using the independent-samples t test or Mann–Whitney U test for continuous variables, and the χ² test or Fisher’s exact test for categorical variables.

Using the failure mode as the unit of analysis, the 11 paired initial and residual RPN summaries were compared using the Wilcoxon signed-rank test. Individual failure modes were summarized descriptively and were not subjected to separate hypothesis tests.

For descriptive setup-error analysis, patient-level median absolute deviations were calculated from fraction-level CBCT couch correction values and compared between groups using the Mann–Whitney U test. To account for repeated fraction-level measurements within patients, linear mixed-effects models were fitted for each setup dimension, with management group as a fixed effect and patient identity as a random intercept. Effect estimates were reported as β coefficients with 95% confidence intervals.

Workflow-related adverse events were analyzed at the patient-treatment-course level. Because of the small number of events, Fisher’s exact test was used for the primary comparison, and relative risk (RR) with 95% CI was reported. All tests were two-sided, and *p* < 0.05 was considered statistically significant.

## 3 Results

### 3.1 Changes in risk priority numbers

As shown in **Table 4**, residual RPN values were numerically lower than initial RPN values for all 11 high-risk failure modes. Using the failure mode as the unit of analysis, the 11 paired initial and residual RPN summaries were compared using the Wilcoxon signed-rank test. The overall median residual RPN was significantly lower than the initial RPN [39.0 (26.0–46.5) vs 177.5 (165.0–209.0); Z = −2.934, *p* = 0.003]. Individual failure modes were summarized descriptively and were not subjected to separate hypothesis tests. The corresponding initial and residual O, S, and D distributions for each failure mode are reported in **Supplementary Table 1**.

**Table 4:** Comparison of pre- and post-intervention RPN scores for high-risk failure modes.

Workflow phase	Operational step	Failure mode	Initial RPN, median (IQR)	Residual RPN, median (IQR)	*Z*	*P* value
Immobilization	Mold fabrication	Inconsistent posture	213.0 (184.5–224.0)	30.0 (17.5–48.0)	–	–
	Mold fabrication	Mold shrinkage/deformation	174.0 (168.0–213.5)	39.0 (15.5–43.5)	–	–
	Isocenter marking	Inconsistent marking phase	205.0 (198.0–228.0)	45.0 (40.5–51.8)	–	–
CT Simulation	Contrast-enhanced CT	Contrast injection affects posture	177.5 (168.0–199.5)	48.0 (36.0–49.5)	–	–
	CT scanning	Irregular breathing	192.0 (175.5–201.0)	48.0 (39.5–54.0)	–	–
	CT reconstruction	Incorrect 4D images	142.0 (126.0–151.8)	26.0 (15.5–42.0)	–	–
Verification & Delivery	Information verification	Plan/prescription mismatch	165.0 (159.0–193.0)	26.0 (15.5–42.0)	–	–
	Information verification	Inconsistent reference images	164.0 (155.0–184.0)	26.0 (15.5–42.0)	–	–
	CBCT	Registration misalignment	213.0 (191.3–224.0)	48.0 (17.5–54.0)	–	–
	Delivery	Inappropriate gating settings	165.0 (159.0–193.0)	45.0 (35.0–49.5)	–	–
	Delivery	Intra-fraction motion	220.0 (180.0–243.0)	16.0 (12.8–22.5)	–	–
Overall median	–	–	177.5 (165.0–209.0)	39.0 (26.0–46.5)	-2.934	0.003

RPN values for each failure mode are presented as median (IQR) across the eight raters. RPN was calculated separately for each rater as O × S × D. Using the failure mode as the unit of analysis, the 11 paired initial and residual RPN summaries were compared using the Wilcoxon signed-rank test. Individual failure modes were not tested separately. Detailed pre- and post-intervention O, S, D, and RPN summaries and rater-level values are provided in **Supplementary Table 1** (**Supplementary Table S1**, Initial_ratings, and Residual_ratings worksheets). D, detectability; IQR, interquartile range; O, occurrence; RPN, risk priority number; S, severity.

### 3.2 Comparison of setup errors

A total of 374 fraction-level CBCT registrations from 63 patients in the control group and 388 registrations from 65 patients in the FMEA-guided group were analyzed. After aggregation into patient-level median absolute deviations, the FMEA-guided group showed smaller translational and rotational setup deviations than the control group. The median Vrt, Lng, and Lat deviations were 0.12, 0.13, and 0.14 cm in the FMEA-guided group, compared with 0.19, 0.21, and 0.21 cm in the control group. Rotational deviations were also lower in the FMEA-guided group (**Table 5**).

**Table 5:** CBCT-derived six-dimensional setup deviations and linear mixed-effects model results.

Dimension	Unit	Control group (n = 63; 374 CBCTs)	FMEA-guided group (n = 65; 388 CBCTs)	Patient-level P value	β, FMEA-guided vs control	95% CI	Mixed-model P value
Translational deviations							
Vertical (Vrt/Z)	cm	0.19 (0.13–0.27)	0.12 (0.08–0.18)	<0.001	-0.064	-0.098 to -0.031	<0.001
Longitudinal (Lng/Y)	cm	0.21 (0.12–0.29)	0.13 (0.08–0.25)	0.002	-0.069	-0.112 to -0.026	0.002
Lateral (Lat/X)	cm	0.21 (0.14–0.25)	0.14 (0.10–0.21)	0.008	-0.056	-0.086 to -0.026	<0.001
Rotational deviations							
Rotation (Rtn)	°	0.40 (0.28–0.60)	0.30 (0.10–0.60)	0.048	-0.145	-0.266 to -0.025	0.018
Pitch	°	0.40 (0.20–0.65)	0.30 (0.10–0.50)	0.029	-0.163	-0.257 to -0.068	<0.001
Roll	°	0.50 (0.30–0.93)	0.30 (0.20–0.50)	0.002	-0.298	-0.415 to -0.181	<0.001

Patient-level values are presented as median (IQR) of absolute fraction-level CBCT couch correction values. Mixed-effects models were fitted using all fraction-level records, with group as a fixed effect and patient identity as a random intercept. Negative β values indicate smaller setup deviations in the FMEA-guided group compared with the control group. CI, confidence interval; CBCT, cone-beam computed tomography; FMEA, failure mode and effects analysis; IQR, interquartile range.

In fraction-level mixed-effects models accounting for within-patient clustering, the FMEA-guided group remained significantly associated with smaller deviations across all six dimensions. The β coefficients were −0.064 cm for Vrt, −0.069 cm for Lng, −0.056 cm for Lat, −0.145°for Rtn, −0.163°for Pitch, and −0.298°for Roll, all favoring the FMEA-guided group.

### 3.3 Incidence of adverse events

Workflow-related adverse events were recorded in 7 of 63 patients in the control group and 2 of 65 patients in the FMEA-guided group. Using the patient treatment course as the denominator, the incidence decreased numerically from 11.11% to 3.08%; however, this difference did not reach statistical significance using Fisher’s exact test (RR = 0.277, 95% CI: 0.060–1.282; *p* = 0.093). Event categories are summarized descriptively in **Table 6**.

**Table 6:** Workflow-related adverse events in the two study cohorts.

Outcome/event type	Control group (n=63)	FMEA-guided group (n=65)	RR (95% CI)	P value
Any workflow-related adverse event	7/63 (11.11)	2/65 (3.08)	0.277 (0.060–1.282)	0.093
Event category details				
Defective molds	3	2	–	–
CT reconstruction errors	2	0	–	–
Treatment plan information error	1	0	–	–
Target volume shift	1	0	–	–

The primary comparison was performed at the patient-treatment-course level using Fisher’s exact test because of the small number of events. Relative risk was calculated for the FMEA-guided group versus the control group. Event categories are summarized descriptively. CI, confidence interval; FMEA, failure mode and effects analysis; RR, relative risk.

## 4 Discussion

SBRT has become a standard treatment option for early-stage NSCLC and selected pulmonary metastases, owing to its ability to deliver highly conformal ablative doses within a limited number of fractions (10, 11). However, the steep dose gradients and narrow treatment margins inherent to lung SBRT impose rigorous requirements on geometric accuracy, respiratory-motion management, image guidance, and multidisciplinary workflow consistency (11, 12). Conventional radiotherapy QC has traditionally emphasized physics-based quality assurance, including equipment performance testing and dosimetric verification (13). Although these procedures remain essential, they may not fully capture process-related vulnerabilities arising from patient immobilization, respiratory coaching, CT simulation, image registration, information transfer, and treatment delivery (8, 14). In the present study, we implemented an FMEA-guided quality-improvement workflow across the lung SBRT process and evaluated its association with process-risk indicators, CBCT-derived setup deviations, and recorded workflow-related adverse events. The revised results showed that residual RPN values were lower after intervention, CBCT-derived six-dimensional setup deviations were smaller in the FMEA-guided group, and workflow-related adverse events decreased numerically but did not reach statistical significance after exact testing.

The present methodology builds upon the clinical adaptation of the AAPM TG-100 framework within the specific context of lung SBRT (8). The highest-risk failure modes identified in this study, including intra-fraction motion, CBCT registration misalignment, and inconsistent patient posture, are consistent with the key vulnerabilities of SBRT workflows reported in previous radiotherapy risk-assessment studies (15, 16). Importantly, the value of this study does not lie solely in the theoretical calculation of RPN, but in the attempt to close the FMEA loop by linking lung SBRT-specific high-risk nodes to targeted corrective measures and measurable process endpoints. In contrast to conventional general-purpose QC checklists, the FMEA process allowed prioritization of workflow nodes that were particularly relevant to lung SBRT, especially those related to respiratory reproducibility, immobilization stability, online image registration, and treatment-verification consistency (17, 18). Therefore, the intervention should be interpreted as an FMEA-guided quality-improvement bundle rather than FMEA alone.

During the risk identification phase, respiration-related and verification-related processes showed high initial RPN values. Lung SBRT is uniquely affected by tumor motion, breathing irregularity, 4DCT reconstruction quality, and the reproducibility of respiratory patterns between simulation and treatment (11, 19). Patients with lung cancer may have impaired pulmonary function, anxiety, coughing, or difficulty maintaining regular breathing, all of which can increase uncertainty during simulation and delivery (20). Inadequate respiratory coaching may lead to irregular 4DCT reconstruction, inaccurate ITV generation, and inconsistent CBCT registration (20–22). Similarly, inappropriate registration strategies or insufficient manual review after automatic registration may propagate geometric uncertainty into treatment delivery. These observations support the need for disease-specific risk management rather than direct application of generic radiotherapy QC procedures.

To mitigate these high-risk failure modes, a multidimensional FMEA-guided quality-improvement strategy was implemented, including process standardization, specialized staff training, respiratory coaching, dual verification, immobilization optimization, gating-threshold management, and enhanced equipment QC. Because these corrective measures were implemented as an integrated workflow bundle, the present study cannot isolate the independent contribution of each individual component. Nevertheless, the intervention was designed according to the risk priorities identified by the FMEA team. For example, inconsistent posture and immobilization-related risks were addressed by standardized SBRT immobilization procedures, improved mold testing, and therapist training (23). Respiratory-motion-related risks were addressed by structured patient education, respiratory training records, and gating-threshold verification (23, 24). Image-registration and information-transfer risks were addressed by manual review after automatic registration, dual-person verification, and standardized reference-image selection (25). This closed-loop process may explain the observed improvement in workflow consistency and CBCT-derived setup reproducibility.

Setup error is an objective and clinically relevant surrogate for evaluating the geometric reproducibility of SBRT delivery (23). In the revised analysis, Patient-level median absolute couch correction values were smaller in the FMEA-guided group across all translational and rotational dimensions. Furthermore, fraction-level linear mixed-effects models, which accounted for repeated CBCT measurements within patients by including patient identity as a random intercept, confirmed that the FMEA-guided group was associated with smaller setup deviations in Vrt, Lng, Lat, Rtn, Pitch, and Roll directions. These findings suggest that the optimized workflow was associated with improved setup reproducibility. The improvement may be related to more consistent immobilization, more standardized respiratory coaching, and stricter manual review of CBCT registration before treatment delivery (23, 24). Given the steep dose gradients and narrow treatment margins of lung SBRT, more reproducible six-dimensional alignment may help support consistent margin application and safer workflow execution (26, 27). However, the present study did not directly evaluate delivered-dose reconstruction, PTV coverage, OAR dose, or clinical toxicity; therefore, the dosimetric and clinical impact of these geometric improvements requires further investigation.

Workflow-related adverse events were also evaluated as process safety indicators, with Fisher’s exact test adopted for revised patient-treatment-course-level comparisons. Workflow-related adverse events were recorded in 7 of 63 patients in the control group and 2 of 65 patients in the FMEA-guided group, corresponding to a numerical decrease from 11.11% to 3.08%. However, this difference did not reach statistical significance (RR = 0.277, 95% CI: 0.060–1.282; *p* = 0.093). The lower number of events in the FMEA-guided group is encouraging and may reflect improved process control, but the small event count limits statistical power. In addition, because the control cohort was retrospectively reviewed whereas the intervention cohort was prospectively monitored, differences in event-detection intensity and reporting behavior may have influenced the observed event rates. This concern is particularly relevant in radiotherapy safety studies, where incident learning and reporting systems may be affected by institutional culture, staff awareness, and reporting behavior (5).

An additional strength of FMEA is that it can identify low-frequency but high-severity risks that may be underestimated by retrospective incident review alone. For instance, treatment-plan information discrepancies or incorrect reference-image selection may occur infrequently, but their potential consequences in lung SBRT can be serious because of steep dose gradients and limited margins (11, 12). By jointly considering occurrence, severity, and detectability, FMEA provides a structured method to prioritize preventive action before patient harm occurs (8). Nevertheless, RPN scoring remains semi-quantitative and partly dependent on expert judgment. Although the initial ratings showed generally good inter-rater reliability, agreement was lower for some residual-risk components, particularly occurrence. This lower agreement may partly reflect the restricted range of post-intervention scores, although evaluator-related variability cannot be excluded. Moreover, residual risk was reassessed by the same unblinded team after implementation of the corrective measures. Therefore, the observed reduction in RPN should be interpreted as a change in a process-risk indicator rather than as an independent clinical endpoint. Future studies may consider modified FMEA approaches, including fuzzy or multi-criteria decision-making models, to reduce expert-dependent bias in risk prioritization (25).

Several limitations should be acknowledged. First, this was a single-center historical-control study; therefore, time-dependent factors such as staff experience, workflow familiarity, patient coaching, and clinical vigilance could not be fully excluded despite stable equipment and a stable multidisciplinary team. Second, the intervention was an FMEA-guided quality-improvement bundle rather than FMEA alone, and the independent contribution of each corrective measure could not be isolated. Third, workflow-related adverse events were uncommon, and their numerical reduction did not reach statistical significance after Fisher’s exact testing; differences in event ascertainment between retrospective and prospective periods may also have affected reporting. Fourth, residual RPN reassessment was not blinded and may have been influenced by evaluator expectations. Finally, long-term clinical outcomes were not evaluated. Prospective multicenter studies with standardized event reporting, independent risk reassessment, and clinical-outcome follow-up are needed to validate this workflow model.

## 5 Conclusion

Implementation of an FMEA-guided quality-improvement workflow in lung SBRT was associated with reduced residual RPN scores and smaller CBCT-derived six-dimensional setup deviations. Workflow-related adverse events decreased numerically but did not reach statistical significance after exact testing. These findings support the practical value of closed-loop proactive risk management for lung SBRT workflow optimization, while prospective multicenter studies with delivered-dose and clinical-outcome endpoints are needed for further validation.

## Data Availability

The raw data supporting the conclusions of this article will be made available by the authors, without undue reservation.
